# Dysregulation of Intestinal Microbiota Elicited by Food Allergy Induces IgA-Mediated Oral Dysbiosis

**DOI:** 10.1128/IAI.00741-19

**Published:** 2019-12-17

**Authors:** Shohei Matsui, Hideo Kataoka, Jun-Ichi Tanaka, Mariko Kikuchi, Haruka Fukamachi, Hirobumi Morisaki, Hitomi Matsushima, Kenji Mishima, Shoji Hironaka, Takashi Takaki, Nobuo Okahashi, Yasubumi Maruoka, Hirotaka Kuwata

**Affiliations:** a Department of Oral Microbiology, Showa University, Tokyo, Japan; b Department of Oral Microbiology, Division of Oral Infections and Health Sciences, Asahi University, Gifu, Japan; c Department of Special Needs Dentistry, Division of Community-Based Comprehensive Dentistry, Showa University, Tokyo, Japan; d Department of Oral Diagnostic Sciences, Division of Pathology, Showa University, Tokyo, Japan; e Department of Pediatric Dentistry, Showa University, Tokyo, Japan; f Department of Special Needs Dentistry, Division of Hygiene and Oral Health, Showa University, Tokyo, Japan; g Division of Electron Microscopy, Showa University, Tokyo, Japan; h Center for Frontier Oral Science, Osaka University Graduate School of Dentistry, Suita, Osaka, Japan; Georgia Institute of Technology School of Biological Sciences

**Keywords:** *Citrobacter*, food allergy, microbiota

## Abstract

Food allergy is a life-threatening response to specific foods, and microbiota imbalance (dysbiosis) in gut is considered a cause of this disease. Meanwhile, the host immune response also plays an important role in the disease. Notably, interleukin 33 (IL-33) released from damaged or necrotic intestinal epithelial cells facilitates IL-2-producing CD4 helper T (Th2) responses. However, causal relationships between the gut and oral dysbiosis and food allergy remain unknown. In this study, we analyzed effects of gut and oral dysbiosis on development of food allergy. A murine model of food allergy was established via ovalbumin (OVA) injection in BALB/c mice. Viable fecal bacteria were identified using matrix-assisted laser desorption ionization–time of flight mass spectrometry (MALDI-TOF MS). *il33* expression in colon-26 mouse colon cells stimulated by isolated fecal bacteria was quantified by real-time PCR. Intestinal T cells from the mice were analyzed by flow cytometry. Salivary IgA levels were quantified by enzyme-linked immunosorbent assay (ELISA), and IgA-bound oral bacteria were detected by flow cytometry. Among fecal bacteria, the abundance of *Citrobacter* sp. increased in the feces of allergic mice and induced *il33* expression in colon-26 cells. Orally administered Citrobacter koseri JCM1658 exacerbated systemic allergic symptoms and reduced intestinal Th17 cells. Salivary IgA and IgA-bound oral bacteria increased in the allergic mice. Based on the results described above, food allergy induced both gut and oral dysbiosis. *Citrobacter* sp. aggravated allergy symptoms by inducing IL-33 release from intestinal epithelial cells.

## Abstract (precis)

Food allergy is a life-threatening response to specific foods, and microbiota imbalance (dysbiosis) in gut is considered a cause of this disease. Meanwhile, the host immune response also plays an important role in the disease. Notably, interleukin 33 (IL-33) released from damaged or necrotic intestinal epithelial cells facilitates IL-2-producing CD4 helper T (Th2) responses. However, causal relationships between the gut and oral dysbiosis and food allergy remain unknown.

## INTRODUCTION

Food allergy is a life-threatening anaphylactic response to foods. Its prevalence is increasing, particularly in developed countries (1). Gastrointestinal immune system dysfunction is considered to induce food allergy, and the intestinal microbiota play important roles in this dysfunction (2). A recent report revealed that clostridia, commensal bacteria indigenous to the murine gastrointestinal tract, induce regulatory T cell accumulation in the mouse colonic lamina propria while decreasing ovalbumin (OVA)-specific IgE production in the sera of OVA-sensitized mice (3). Clostridia alter the composition of the commensal gastrointestinal microbiota, and the clostridium-containing gastrointestinal microbiota suppress interleukin 2 (IL-2)-producing CD4 helper T (Th2) cytokine production and allergen accession by inducing IL-22 production in mouse serum (4). In addition, orally administered Lactobacillus brevis HY7401 induces Th1 cytokine production and inhibits Th2 cytokine production (5). These reports demonstrate that the intestinal bacteria comprising the gastrointestinal microbiota are capable of affecting host Th2 responses. However, the detailed relationships between food allergies and the gastrointestinal microbiota remain to be elucidated. Conversely, the relationship between the oral microbiota composition, which influences composition of the gastrointestinal microbiota, and food allergy symptoms remains completely unknown, even though the effects of orally administered bacteria have been investigated (5).

IL-33, a member of the IL-1 family of cytokines, is produced by epithelial cells, keratinocytes, fibroblasts, and other immune cells (6). IL-33 induces the expression of cytokine-encoding genes in Th2 cells and activates MAP kinases in mast cells by binding to the unique IL-33-specific receptor ST2 (6–8). Upon the infiltration of damaged epithelial cells, bacterial pathogens and commensal bacteria induce host production of IL-33, which in turn activates Th2 cells, basophils, mast cells, and group 2 innate lymphoid cells (7). These results illustrate the crucial role of IL-33 in inducing Th2 responses in the gastrointestinal tract, suggesting a causal relationship between IL-33 induction and food allergy. Therefore, the IL-33-inducing activity of intestinal bacteria influences the severity of food anaphylaxis.

In the present study, we identified an intestinal bacterium that specifically accumulates in the gastrointestinal tract in a mouse model of food allergy. We then examined the *il33*-inducing activity of this bacterium. Concurrently, we also compared the composition of the oral microbiota between food-allergic mice and control mice. Our results demonstrated that intestinal microbiota alteration exacerbates food anaphylaxis, confirming the food allergy-inducing ability of gastrointestinal commensal bacteria. Interestingly, our results suggest that food allergy also induces oral dysbiosis.

## RESULTS

### Establishment of a murine model of food allergy (OVA/alum mice).

**FIG 1:**
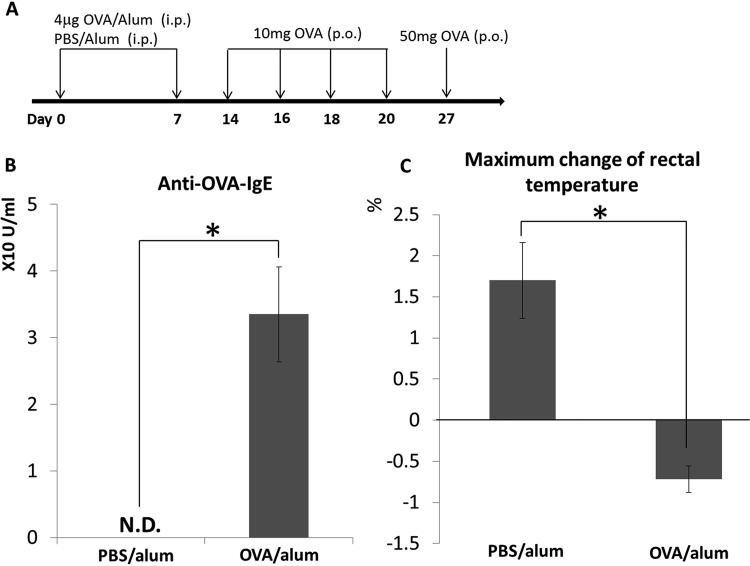
Establishment of food allergy mouse model. Timeline of ovalbumin (OVA) sensitization and the induction of food allergy in mice (A). The production of OVA-specific IgE antibodies in the sera of both groups of mice was measured by enzyme-linked immunosorbent assay (ELISA) (B). OVA-specific food allergy symptoms were assessed by measuring the maximum change in rectal temperature after the oral administration of 50 mg of OVA. Percentage changes, based on values before treatment, after the oral administration of 50 mg of OVA are indicated (C). Results are presented as the mean ± standard deviation (SD) (*n* = 3) and represent three independent experiments. N.D., not detected; p.o., oral(ly). *, *P* < 0.05.

A schematic of the establishment of the mouse model of food allergy is presented in Fig. 1A. Serum concentrations of OVA-specific IgE were significantly higher in OVA/alum mice than in phosphate-buffered saline (PBS)/alum mice, demonstrating that OVA/alum mice were sensitized to OVA (Fig. 1B). Oral (p.o.) administration of 50 mg of OVA elicited a significant decrease in rectal temperature in the OVA/alum group compared with that in the control group (Fig. 1C). As decreased rectal temperature after p.o. administration of antigen is a representative symptom of food allergy (9–11), our results demonstrated the establishment of a murine model of food allergy. In addition, OVA/alum mice showed significant systemic symptoms, such as diarrhea (data not shown), which is a prominent marker for food anaphylaxis as referred to in guidelines of the European Academy of Allergy and Clinical Immunology (EAACI) and the American Academy of Allergy and Asthma and Immunology (AAAAI) (12, 13).

### *Citrobacter* sp. significantly propagated in the gastrointestinal tract of OVA/alum mice.

**FIG 2:**
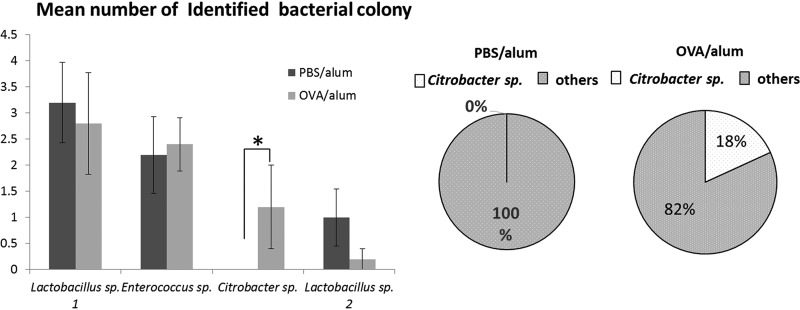
Identification of fecal bacteria of ovalbumin (OVA)-specific food-allergic mice. Feces from food-allergic (OVA/alum) and control (phosphate-buffered saline [PBS]/alum) mice were collected and analyzed using a Vitek MALDI-TOF MS system. The numbers of identified fecal bacteria derived from each group of mice (OVA/alum group, OVA/alum 1 to OVA/alum 5; PBS/alum group, PBS/alum 1 to PBS/alum 5) are indicated in the left bar graph. Results are presented as the mean ± standard error (SE) (*n* = 5). *, *P* < 0.05. The presence of *Citrobacter* sp. in all fecal samples from both groups of mice is indicated in the circle charts on the right.

To identify intestinal bacteria that specifically accumulated in the gastrointestinal tract of food-allergic mice, we identified viable bacteria in the feces of both groups of mice by using the Vitek system, which uses matrix-assisted laser desorption ionization–time of flight mass spectrometry (MALDI-TOF MS) to identify bacterial colonies that form on blood agar plates inoculated with feces of mice. This analysis made it possible to compare the quantities of viable bacteria between food-allergic mice and healthy control mice. Use of this method revealed the allergy-inducing ability of intestinal bacteria more clearly than other methods that used samples containing dead bacteria or fragments of bacterial components, because host cells respond to bacterial infiltration or bacterial metabolites to elicit immune responses. Our system identified four species of bacteria—all of which are frequently found in the intestines of mice—with 100% reliability. The current analysis revealed that the density of *Citrobacter* sp. was selectively elevated in the feces of OVA/alum mice compared with that in control mice (Fig. 2).

### *Citrobacter* sp. induces IL-33 expression in a colon cell line.

**FIG 3:**
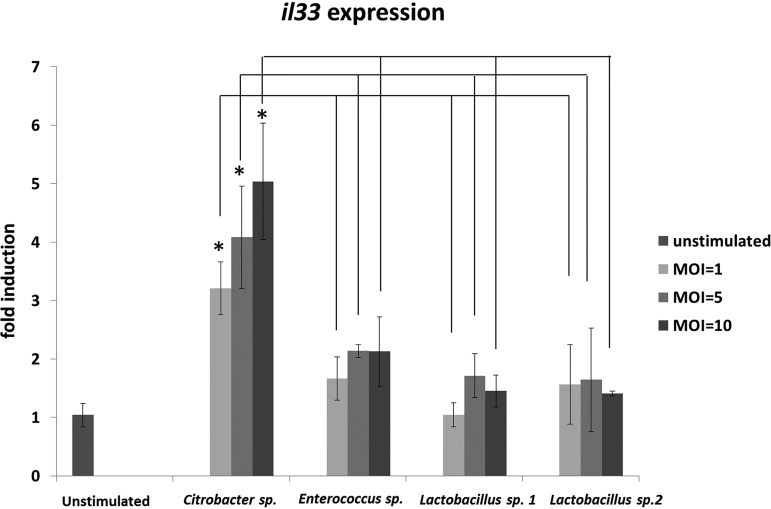
*In vitro* interleukin 33 gene (*il33*) expression following stimulation by fecal bacteria isolated from ovalbumin (OVA)-induced allergic mice. Cells from colon-26, a mouse colon epithelial cell line, were stimulated by four species of viable bacteria isolated from the feces of OVA-allergic mice. *il33* expression levels in each sample were normalized to those of a housekeeping gene (*gapdh*) and expressed as fold changes relative to levels in a nonstimulated sample. Results are presented as the mean ± SD (*n* = 3) and represent three independent experiments. *, *P* < 0.05.

Recent reports revealed that IL-33 plays an important role in allergic responses (14, 15), including the exacerbation of food-induced anaphylaxis in the gastrointestinal tract (9). We therefore examined whether fecal bacteria, including *Citrobacter*, induce *il33* expression in gastrointestinal epithelial cells under *in vitro* conditions. Cells from a mouse colon epithelial cell line, colon-26, were stimulated by four species of bacteria, which were isolated from the feces of mice and identified by the Vitek MALDI-TOF MS system with 100% reliability. Among the four tested species of fecal bacteria, only *Citrobacter* sp. induced *il33* expression in a dose-dependent manner (Fig. 3).

### Orally administered *Citrobacter* exacerbates an allergic symptom.

**FIG 4:**
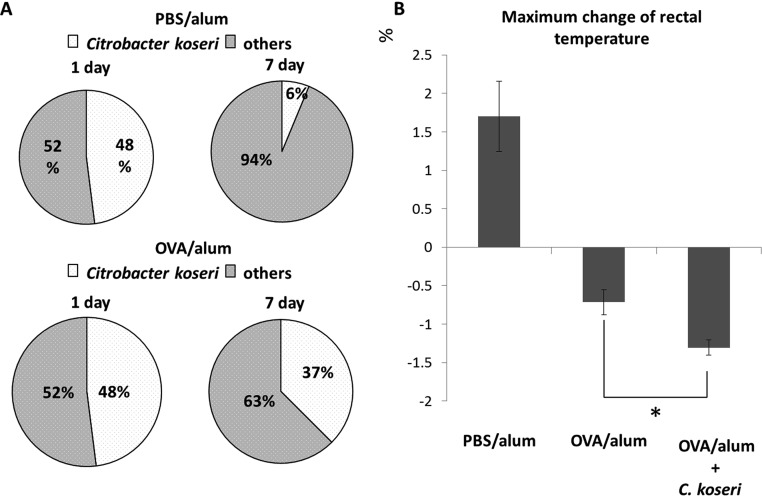
Exacerbation of allergy symptoms by oral administration of *Citrobacter*. After 1 week of antibiotic-mediated depletion of the endogenous microbiota, ovalbumin (OVA)/alum and phosphate-buffered saline (PBS)/alum mice were orally administered Citrobacter koseri (1 × 10^9^ CFU per animal). Feces were collected 1 and 7 days after administration, and the resulting bacterial isolates were identified to the species level using a Vitek MALDI-TOF MS system. The occupancy rate of *C. koseri* among the identified fecal bacteria was defined as the survival rate compared with that of the inoculum (A). The severities of OVA-specific allergic responses were compared by determining anaphylactic responses (assessed as the maximum change in rectal temperature following OVA challenge) in OVA/alum mice after the oral administration of *C. koseri* (B). *, *P* < 0.05.

**FIG 5:**
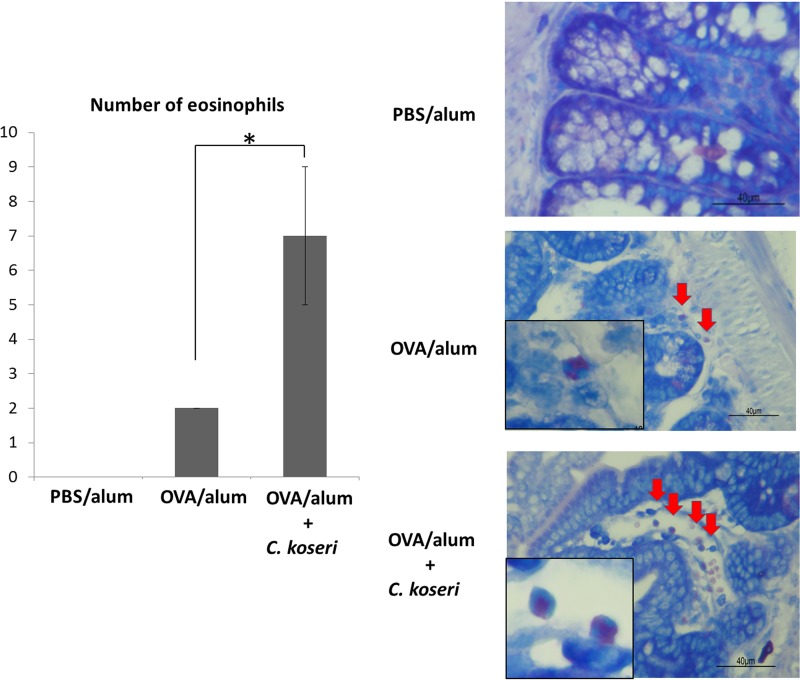
Numbers of eosinophils that infiltrated the small intestines of ovalbumin (OVA)/alum mice after the oral administration of Citrobacter koseri. Numbers of eosinophils that infiltrated the small intestine were visualized using May-Grünwald-Giemsa staining and counted in a high-power field at ×400 magnification. Tissue images are shown on the right. Arrows indicate eosinophils. Left graph shows the mean numbers of eosinophils calculated per three high-power fields.

**FIG 6:**
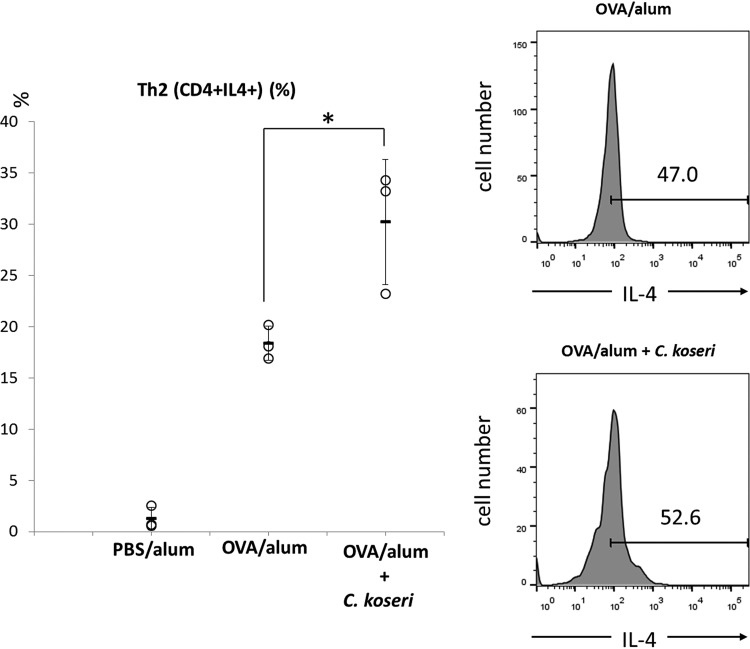
Induction of Th2 cells in the small intestines of ovalbumin (OVA)/alum mice after the oral administration of Citrobacter koseri. Lymphocytes were obtained from the small intestinal lamina propria of OVA/alum mice and after the oral administration of *C. koseri*. The induction of CD4^+^ IL-4^+^ (Th2) cells was analyzed by flow cytometry. In the left graph, circles represent separate mice, and lines denote means ± SDs (*n* = 3). The right histograms present the mean fluorescent intensities of IL-4^+^ cells in OVA/alum and *C. koseri*-administered OVA/alum mice. *, *P* < 0.05.

To further confirm the augmentation of allergic responses by *Citrobacter*, we examined the influence of gastrointestinal microbiota (comprising predominantly *Citrobacter*) on the allergic responses induced by a food antigen. The analysis revealed that p.o.-administered C. koseri survived longer in the feces of OVA/alum mice than in the feces of PBS/alum mice (Fig. 4A). Using these two groups of mice, we next examined whether orally administered *C. koseri* exacerbated the allergic response. Monitoring of rectal temperatures demonstrated that p.o.-administered OVA elicited a significantly lower rectal temperature in *C. koseri*-inoculated OVA/alum mice than it did in OVA/alum mice (Fig. 4B). In addition, histological observation of small intestinal tissue demonstrated that p.o. administration of *C. koseri* significantly increased the number of eosinophils in OVA/alum mice (Fig. 5). Furthermore, an examination of the proportion of Th2 lymphocytes induced in the small intestinal lamina propria revealed that p.o. administration of *C. koseri* yielded an increased proportion of CD4^+^ IL-4^+^ lymphocytes (Th2 lymphocytes) in OVA-specific allergy mice (Fig. 6).

**FIG 7:**
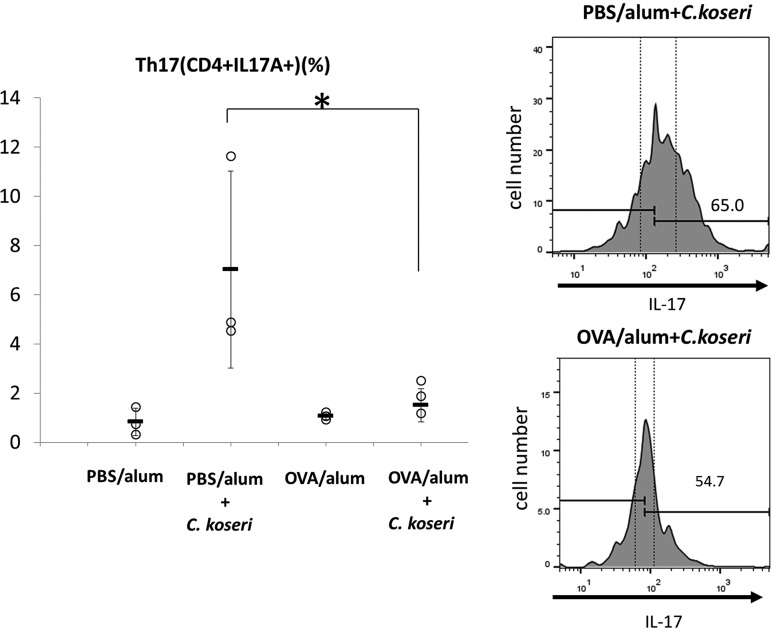
Induction of Th17 cells in the small intestines of mice after the oral administration of Citrobacter koseri. Lymphocytes were obtained from the small intestinal lamina propria of ovalbumin (OVA)/alum and phosphate-buffered saline (PBS)/alum mice after the oral administration of *C. koseri*. Inductions of CD4^+^ IL-17^+^ (Th17) cells were analyzed by flow cytometry. Histograms on the right present the mean fluorescent intensities of CD4^+^ IL-17^+^ (Th17) cells in total lymphocytes derived from each group of mice. In the graph on the left, circles represent separate mice, and lines denote means ± SDs (*n* = 3). The data are representative of three independent experiments. *, *P* < 0.05.

Recent studies reported that several intestinal bacteria—including Citrobacter rodentium, which is closely related to *C. koseri*—induce the accumulation of Th17 cells in the intestinal lamina propria (16, 17). Consistent with those reports, we observed that p.o.-administered *C. koseri* strongly induced Th17 cell accumulation in the intestinal lamina propria of PBS/alum mice but not in OVA/alum animals (Fig. 7). These results are consistent with the known role of Th17 cells in protecting the intestinal mucosa against exogenous pathogenic bacteria, including C. rodentium (16, 17), and they may explain why orally administered *C. koseri* is more readily fixed in the gastrointestinal microbiota of OVA/alum mice than in that of PBS/alum mice.

### Allergic symptoms changed the composition of the oral microbiota.

**FIG 8:**
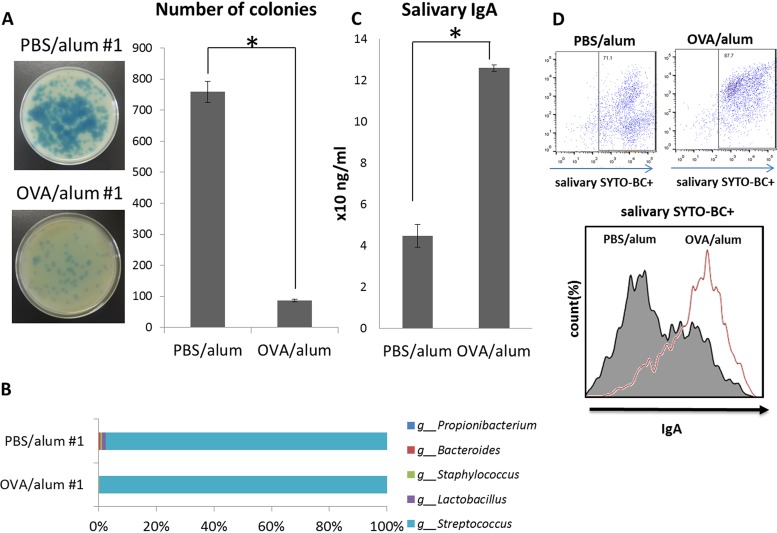
Quantification of hydrogen peroxide-producing bacteria and salivary IgA in the oral cavity of ovalbumin (OVA) allergic mice. Saliva was collected from OVA allergic mice (OVA/alum 1 to 3) and control mice (phosphate-buffered saline [PBS]/alum 1 to 3) and inoculated onto three Prussian blue plates. After an overnight anaerobic incubation, hydrogen peroxide-producing bacteria formed colonies with blue halos. The plates of OVA/alum 1 and PBS/alum 1 are presented (left). The right graph shows the mean numbers of colonies with blue halos for the OVA/alum and PBS/alum groups (A). The percentages of detected genera of bacteria in both OVA/alum 1 and PBS/alum 1 are indicated by a horizontal bar graph (B). Saliva was collected from both OVA/alum and PBS/alum mice, and salivary IgA was quantified using an IgA ELISA kit (C). Oral bacteria in the saliva of both groups of mice were stained using SYTO BC and goat anti-mouse IgA-Alexa Fluor 647. The histogram presents the mean fluorescent intensities of IgA-Alexa Fluor 647-positive oral bacteria in SYTO BC-positive oral bacteria from OVA/alum and PBS/alum mice (D). Results are presented as the mean ± SD (*n* = 3), and they represent three independent experiments. *, *P* < 0.05.

Although some causal relationships between food allergy symptoms and the gastrointestinal microbiota were expected, the role of the oral microbiota in food allergy is unknown. We therefore compared the oral microbiota composition of OVA/alum mice with that of PBS/alum mice. In the human oral cavity, it is well known that the oral cavity microbiota is normally dominated by oral streptococci, including Streptococcus sanguinis and Streptococcus gordonii, which explicitly produce hydrogen peroxide (18). In the mouse oral cavity, it has been reported that Lactobacillus spp. also dominate the oral microbiota in addition to Streptococcus spp. (19). *Lactobacillus* spp. are reported to produce hydrogen peroxide (20). As a result, fewer colonies with blue halos were formed on plates inoculated by saliva from OVA/alum mice than on plates exposed to saliva from PBS/alum mice (Fig. 8A and Fig. S1). In addition, 16S rRNA sequencing confirmed the reduced diversity of oral commensal bacteria in OVA/alum mice (Fig. 8B). Furthermore, IgA-coated bacterial counts have been reported to increase in the gastrointestinal tracts of patients with inflammatory bowel disease (IBD) (21). The report suggested that IgA plays a key role in changing the gastrointestinal microbiota composition. In our experiment, salivary IgA levels were significantly higher in the oral cavities of OVA/alum mice than in those of PBS/alum mice (Fig. 8C). The increased salivary IgA content was bounded by oral bacteria (Fig. 8D).

## DISCUSSION

In this study, we demonstrated that intestinal microbiota alteration can exacerbate food anaphylaxis. Intestinal commensal bacteria affect the development of the host gastrointestinal immune system, and antibiotic-induced intestinal microbiota alteration increases host susceptibility to colonization by pathogenic bacteria (22–24). Several commensal bacteria that protect or induce IBD have been reported (22). Indeed, fecal microbiota transplantation has been proposed as an efficient method for treating IBD (25). IBD is widely known to be a representative disorder induced by excessive Th17 responses (26). Th17 responses and Th2 responses are known to operate in a trade-off relationship. These facts suggest that food allergy as an excessive Th2 response can be controlled by commensal bacteria in much the same way that IBD can be controlled by excessive Th17 responses. Both commensal microbiota and specific bacteria have been revealed to provide protection against food allergy (4, 27).

We used a Vitek system to identify intestinal bacteria that were specifically enriched in a murine model of food allergy. The validity of bacterial identification obtained by the Vitek system has been established by Dubois et al. (28). However, causal relationships between food allergy and the composition of the intestinal microbiota remain to be elucidated. In the present study, we identified a *Citrobacter* bacterium that was specifically enriched in the gastrointestinal tract in a mouse model of food allergy. Dysbiosis cannot be defined by changes in only one microorganism. However, *Citrobacter* sp., instead of numerous intestinal bacteria, can act as an indicator of dysbiosis because *Citrobacter* species are among the most frequently encountered bacteria in the intestine (29), and occupancy in intestinal microbiota is strongly influenced by various disorders (30). Although the virulence and pathogenicity of C. rodentium in the gastrointestinal tract have been investigated (31), the food allergy-inducing abilities of *Citrobacter* spp. remain to be demonstrated. C. rodentium is reported to employ a pathogenic mechanism of epithelial attachment and effacement (A/E) (32, 33), whereby this species attaches to the surface of intestinal epithelial cells and induces local destruction (effacement) of these cells. This mechanism triggers reactive inflammation and the induction of IL-17-producing CD4 helper T (Th17) cells and IL-22-producing type 3 innate lymphoid cells (32, 33). IL-33 is an alarmin cytokine that is released after cell or tissue damage (8). As a *Citrobacter* sp. was isolated from the feces of food-allergic mice, this species is presumed to employ some attachment and infiltration mechanisms similar to A/E in C. rodentium. The *Citrobacter* sp. isolated in the present work is thus expected to be capable of disrupting host intestinal epithelial cells, thereby triggering IL-33 production. In the present work, we observed the *il33*-inducing activity of *Citrobacter* sp. isolated from OVA/alum mice. IL-33 is a Th2 response-driving cytokine. We further demonstrated that another *Citrobacter* species, *C. koseri* JCM1658, exacerbated the anaphylactic response when administered p.o. to OVA/alum mice. Because it has been widely recognized that *Citrobacter* spp.—particularly Citrobacter amalonaticus, Citrobacter farmeri, and Citrobacter koseri—indicate a high homology of genes and biochemical qualities (34, 35), we confirmed that both isolated *Citrobacter* species induce allergic responses *in vitro* and that commercial Citrobacter koseri JCM1658 induces allergic responses *in vivo.* These results clearly demonstrated a causal relationship between *Citrobacter* species and food anaphylaxis.

Reports indicate that C. rodentium induces Th17 cells in the gastrointestinal tract and that Th17 cells in turn facilitate the elimination of exogenous pathogenic bacteria, including *Citrobacter* (15, 16). We postulate that attenuation of Th17 cell levels observed in this study can be attributed to Th2-biased responses in OVA/alum mice. Presumably, a trade-off occurred between Th2 and Th17 responses, such that (in the gastrointestinal tracts of OVA/alum mice) the Th2 response predominated over the weakened induction of Th17 cells, permitting the increased induction of Th2 cells. Apparently, the depleted pool of Th17 cells was not sufficient to eliminate *C. koseri* within a week of p.o. administration. The surviving *C. koseri* bacteria presumably then stimulated the intestinal epithelial cells to produce IL-33, which in turn further reinforced the Th2 response in the gastrointestinal tracts of OVA/alum mice. Furthermore, IL-33 can stimulate naive T cells to exhibit Th2 responses but not Th17 responses (36). Such a circular feedback loop is expected to be an exacerbating factor for food allergy symptoms, facilitating further dysbiosis in the gastrointestinal tract. The resulting dynamic, imbalanced composition of the gastrointestinal microbiota has a causal relationship with food allergy symptoms. Thus, in the present study, we demonstrated that intestinal microbiota alteration exacerbates food anaphylaxis and the food allergy-inducing ability of intestinal commensal bacteria.

In addition to microbiota alteration in the intestine, we demonstrated that the oral microbiota is also affected by food allergy. Although no detailed molecular mechanisms have been uncovered, an extended duration of food allergy symptoms changed the composition of the oral microbiota. Repeated stimulation by allergens derived from food can induce Th2 responses in the gastrointestinal tract, and intestinal microbes affected by Th2 responses trigger a vicious circle of systemic dysbiosis and allergic symptoms. This oral dysbiosis is considered the result of Th2 conversion. Atopic B cells activated in the gastrointestinal tract are assumed to migrate through common mucosal immune systems and affect the oral microbiota composition (37). Lactobacillus spp. are reported to be one of the dominant bacteria in the oral cavities of mice (19); these bacteria are known to produce hydrogen peroxide (20). The study results suggested that hydrogen peroxide-producing *Lactobacillus* sp. counts are decreased in the oral cavities of allergic mice, with a similar finding noted in the feces of OVA/alum mice. The similar compositional changes in both the oral and the gastrointestinal microbiota indicated that the microbiota alteration in the intestine induced in food allergy affects the oral microbiota. The importance of intestinal IgA for maintaining the commensal bacteria in the gastrointestinal tract was recently reported (21, 38). In the feces of patients with IBD, colitogenic members of the intestinal microbiota were coated with IgA (21). Oral supplementation of high-affinity and poly-reactive IgA improved the pathological colon phenotype and increased regulatory T cell counts in the colon (38).

The results of the current study suggest that the Th2-biased intestinal condition induced by food allergy allows *Citrobacter* spp. to proliferate in the intestine and that *Citrobacter* spp. aggravate allergic symptoms by inducing IL-33 release from intestinal epithelial cells. In this way, food allergy can be considered to induce microbiota alteration in the intestine．Furthermore, the microbiota alteration in the intestine stimulates IgA induction in the gastrointestinal tract, and the increase in IgA affects the oral microbiota (Fig. S2). Our findings suggest a causal relationship between food allergy as a systemic disease and oral dysbiosis.

## MATERIALS AND METHODS

### Mouse model of food allergy.

Animal experiments were approved by the Institutional Animal Care and Use Committee of Showa University, Japan (approval number 18008). Female BALB/c mice (aged 8 weeks; CLEA Japan, Inc., Tokyo, Japan) were maintained under specific-pathogen-free conditions. These mice were housed in sterilized cages, and they received a standard mouse diet (Certified Diet MF; Oriental Yeast, Tokyo, Japan) and autoclaved water. The mice were sensitized via two intraperitoneal (i.p.) injections (at a 1-week interval) of 4 μg of ovalbumin (OVA; Wako, Osaka, Japan) mixed with 100 μl of aluminum hydroxide gel adjuvant (alum; Invivogen, San Diego, CA, USA). Starting 1 week after the second injection, food allergy was induced via oral (p.o.) administration using a feeding needle of four doses (over the course of 1 week) of 10 mg of OVA dissolved in phosphate-buffered saline (PBS). To elicit allergic responses, mice were p.o. administered 50 mg of OVA dissolved in PBS 1 week after the last 10-mg OVA administration, according to a well-established method for eliciting OVA-specific food allergy in mice (39–41). To evaluate whether a food allergy was elicited, OVA-specific IgE levels in the sera of the mice were measured using a mouse OVA-IgE enzyme-linked immunosorbent assay (ELISA) kit (AKRIE-30; Shibayagi, Gumma, Japan). Systemic anaphylaxis in the mice was evaluated by monitoring for decreased rectal temperature using a weighing environment logger (AD-1687; A and D, Tokyo, Japan).

### Identification of fecal bacteria.

The feces of both OVA/alum mice and PBS/alum mice were suspended in 20 volumes of PBS, inoculated onto plates containing Columbia agar with 5% sheep blood, and cultured anaerobically for 2 days at 37°C. To identify viable bacteria in the feces of the mice, a portion of each fresh colony that developed on each plate was smeared onto a target slide as a sample. These samples were covered with α-cyano-4-hydroxycinnamic acid matrix solution and loaded into a Vitek MALDI-TOF MS system (bioMérieux, Lyon, France). The spectra of the samples were analyzed using the Myla database to identify the species of origin (42). A total of 48 fresh colonies from each group of mice were analyzed.

### mRNA expression.

Colon-26 RCB2657, a mouse colon epithelial cell line, was purchased from the Cell Engineering Division of Riken BioResource Center (Tsukuba, Japan). The cells were cultured in RPMI 1640 medium (Wako, Tokyo, Japan) supplemented with 10% heat-inactivated fetal bovine serum (FBS) at 37°C in a humidified 5% CO_2_ atmosphere. The cells were stimulated by each of four species of feces-extracted bacteria (*Citrobacter* sp., Enterococcus sp., *Lactobacillus* sp. 1, and *Lactobacillus* sp. 2), each at multiplicities of infection of 1, 5, and 10, for 4 h. Total RNA from the cells was isolated using TRIzol reagent (Thermo Fisher Scientific, MA, USA) and quantified spectrophotometrically. Then, 200 ng of total RNA was converted into cDNA using a ReverTra Ace quantitative PCR (qPCR) real-time (RT) kit (Toyobo, Osaka Japan). The primer pairs for mouse *il33* (sense, 5′-GATGGGAAGAAGGTGA-3′; antisense, 5′-TTGTGAAGGACGAAGA-3′) and *gapdh* (sense, 5′-GCACAGTCAAGGCCGAGAAT-3′; antisense, 5′-GCCTTCTCCATGGTGGTGAA-3′) transcripts were used for real-time PCR. Amplification was performed using a Fast SYBR green master mix (Applied Biosystems, CA, USA) and the Step One Plus real-time PCR system (Applied Biosystems). *il33* expression in each sample was normalized to that of *gapdh* (as an internal control) and a nonstimulated cell sample (as a calibrator) using the comparative threshold cycle (*C_T_*) method.

### Oral administration of bacteria.

For antibiotic-mediated depletion of the gastrointestinal microbiota, mice were permitted *ad libitum* access to drinking water containing an antibiotic cocktail consisting of ampicillin (0.1 g/ml; Wako), kanamycin (0.2 g/ml; Wako), and enrofloxacin (120 mg/ml; Bayer, Tokyo, Japan) for 1 week. Citrobacter koseri JCM1658, purchased from the Cell Engineering Division of Riken BioResource Center, was cultured at 37°C under anaerobic conditions in brain heart infusion (BHI) broth. After the gastrointestinal microbiota of the mice had been depleted, they were p.o. administered using a feeding needle 200 μl of 5% NaHCO_3_ in PBS, followed by 1 × 10^9^ CFU of live *C. koseri* JCM1658 suspended in 400 μl of PBS. Changes in the bacterial composition of the gastrointestinal microbiota were evaluated via identification to the species level of fecal bacteria using the aforementioned plating and Vitek methods.

### Flow cytometry.

Mice were anesthetized via i.p. injections of ketamine-xylazine (ketamine, 100 mg/kg body weight; xylazine, 10 mg/kg body weight) and perfused with Hanks’ balanced salt solution (HBSS) containing EDTA for blood removal. The small intestines of the mice were longitudinally opened, washed with PBS, and shaken in HBSS containing 2 mM EDTA for 70 s. The intestines were shredded and shaken in RPMI 1640 containing 4% FBS, 0.2 mg/ml collagenase type 1 (Wako), 0.4 mg/ml dispase 1 (Wako), and 10 μg/ml DNase I (Nippon Gene, Japan) for 30 min at 37°C. Cells in the digested tissue were passed through a cell strainer, and single-cell suspensions were pretreated with brefeldin A (100 ng/ml) for 1 h. The cells were then stained with allophycocyanin-conjugated anti-mouse CD3ε (Becton, Dickinson [BD], NJ, USA), Brilliant Violet 421 (BV-421)-conjugated anti-mouse CD4 (BD), phycoerythrin (PE)-conjugated anti-mouse IL-17A (BD), PE-conjugated anti-mouse IL-17F (BD), and fluorescein isothiocyanate-conjugated anti-mouse CD4 (BD) antibodies. These cells were analyzed using a FACSVerse flow cytometer (BD).

### Histological analysis.

Small intestinal tissues were fixed in 4% paraformaldehyde and embedded in paraffin. Sections were stained via May-Grünwald-Giemsa staining. The numbers of eosinophils were counted under a high-power field at ×400 magnification. The numbers of eosinophils are expressed as the means of counts of three high-power fields.

### Quantification of hydrogen peroxide-producing bacteria in the oral cavity.

Saliva from both OVA/alum and PBS/alum mice was obtained using cotton swabs from the oral cavity, and these cotton swabs were each immersed in 500 μl of PBS. Aliquots (50 μl) of the swab-immersed PBS were inoculated onto a Prussian blue (PB) agar plate to measure hydrogen peroxide production. PB plates were prepared by dissolving 1 g of FeCl3·6H2O, 1 g of potassium hexacyanoferrate(III), 36 g of BHI broth, and 15 g of agar in deionized water (43). Hydrogen peroxide-producing bacteria were visualized by the formation of colonies with a blue halo on the PB plates after anaerobic incubation.

### 16S rRNA sequencing.

Saliva was obtained from OVA/alum and PBS/alum mice as described previously. Total RNA was isolated from swab-immersed PBS samples using an RNeasy minikit (Qiagen, Hilden, Germany). All RNA samples were sent to Hokkaido System Science (Sapporo, Japan), which performed 16S rRNA sequencing.

### Quantification of salivary IgA levels and analysis of IgA-bound oral bacteria.

OVA/alum and PBS/alum mice were anesthetized as described previously. Five minutes after the ketamine-xylazine injection, mice were i.p. injected with 1 mg/kg body weight of pilocarpine hydrochloride (Santen, Osaka, Japan) to stimulate salivary secretion. Saliva was collected from the oral cavity of each mouse using a micropipette. Total IgA in the saliva was quantified using an IgA mouse uncoated ELISA kit (Thermo Fisher Scientific). Collected salivary samples from both groups of mice were centrifuged at 8,000 × *g* to pellet bacteria. The bacterial pellets were resuspended in 660 nM SYTO BC (Thermo Fisher Scientific) in 0.25% bovine serum albumin (BSA)/PBS. After 30 min of incubation on ice, the suspensions were centrifuged at 8,000 × *g*, and bacterial pellets were resuspended in 1.3 μg/ml goat anti-mouse IgA-Alexa Fluor 647 (Southern Biotech, AL, USA) in 0.25% BSA/PBS and incubated for 30 min on ice. After washing, bacterial pellets were resuspended in 0.25% BSA/PBS and analyzed using a FACSVerse flow cytometer (BD).

### Statistical analysis.

The statistical differences were assessed using a two-tailed Student’s *t* test, and homoscedasticity of the data was assumed. *P* values of ≤0.05 were considered significant.

## Supplementary Material

Supplemental file 1
