# Supplementary material for: Dysregulation of Intestinal Microbiota Elicited by Food Allergy Induces IgA-Mediated Oral Dysbiosis
Source: Infect Immun. 2019 Dec 17;88(1):e00741-19. doi: 10.1128/IAI.00741-19 (PMC6921656; doi:10.1128/IAI.00741-19)
Supplement: Supplemental file 1 [file IAI.00741-19-s0001.pdf]

Supplemental figures

Fig. S1

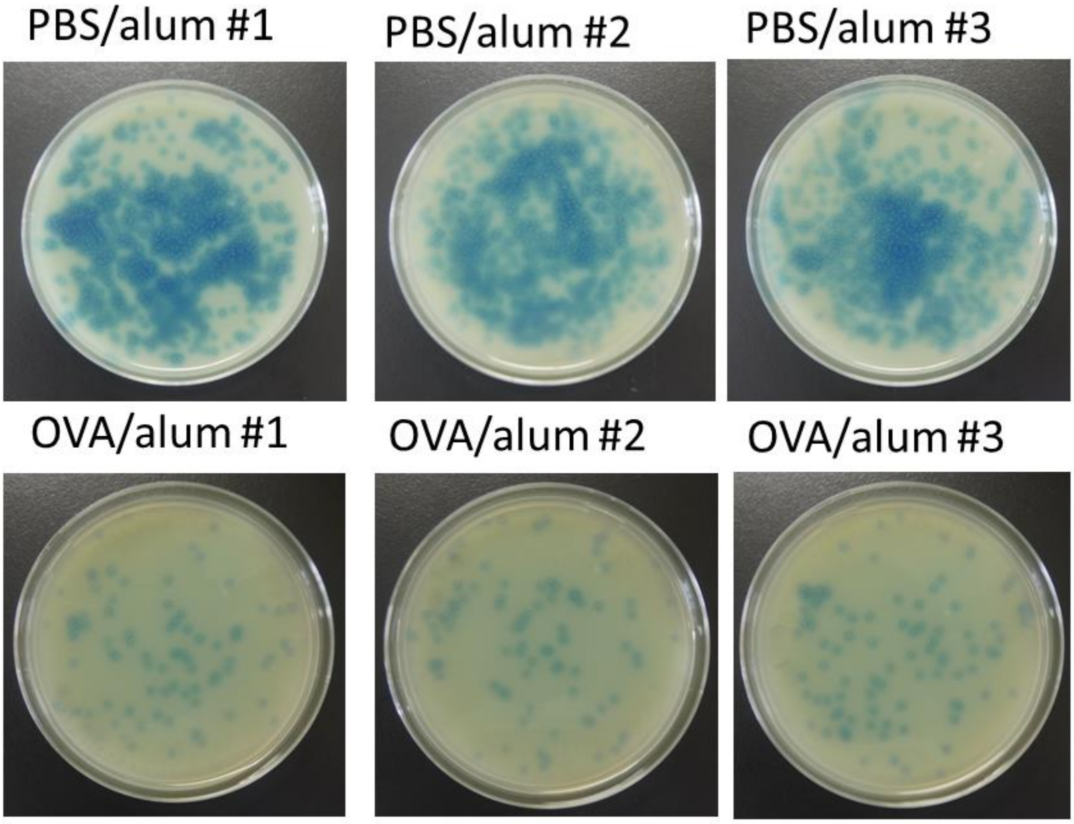

Fig. S2

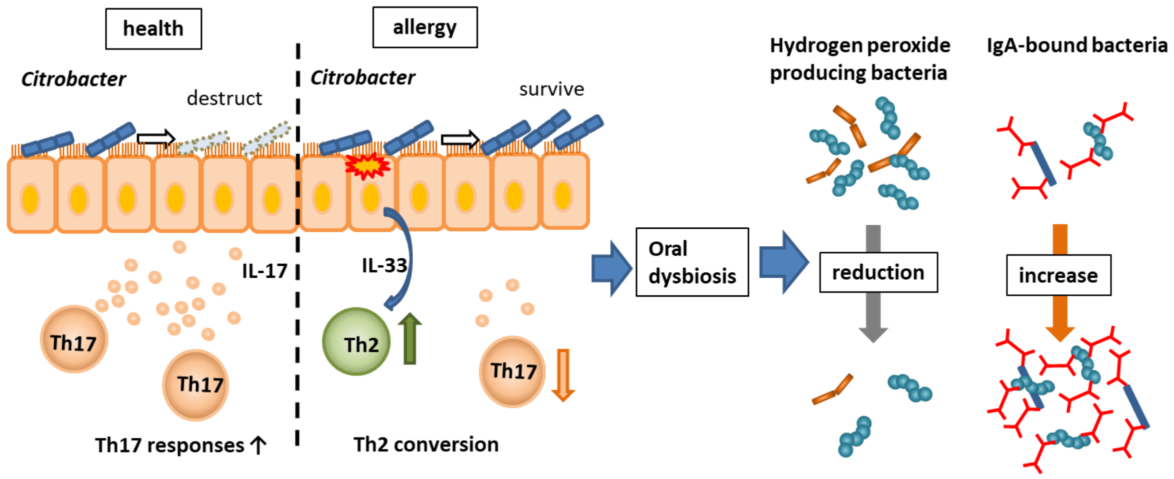
health
allergy
Citrobacter
destruct
Citrobacter
survive
IL-17
IL-33
Th17
Th17
Th2
Th17
Th17 responses ↑
Th2 conversion
Oral dysbiosis
Hydrogen peroxide producing bacteria
IgA-bound bacteria
reduction
increase

Supplemental figure legends

<u>Fig. S1.</u> Each Prussian blue plate inoculated by saliva of OVA allergic and control mice. Saliva of OVA allergic (OVA/alum #1–#3) and control mice (phosphate-buffered saline [PBS]/alum #1–#3) were inoculated onto three Prussian blue plates. After an overnight anaerobic incubation, hydrogen peroxide-producing bacteria formed colonies with blue halos. The plates of OVA/alum #1 and PBS/alum #1 are shown in Fig. 8A as representative data.

<u>Fig. S2.</u> Graphical Abstract of this study
This figure illustrates our finding of this study. Although *Citrobacter spp.* are destructed by Th17 responses in healthy intestine, Th2-biased, Th17-weakend, allergic intestinal condition allows them to proliferate and aggravate allergic symptoms by inducing IL-33 release from intestinal epithelial cells. Further, this dysregulation of intestinal microbiota elicits oral dysbiosis by reducing hydrogen peroxide producing bacteria and stimulating IgA induction.
